# Comparative effectiveness of tirzepatide and DPP-4 inhibitors in type 2 diabetes with heart failure

**DOI:** 10.1177/14791641261467888

**Published:** 2026-07-08

**Authors:** Ibrahim Mortada, Krishna Paul, Michael Chammany, Youstina Abraham, Karthik Devulapally, Aaron W. Lee, Shareef Mansour, Khaled Chatila, Michael C. Boyars, Dietrich Jehle, Thomas A. Blackwell, Hani Jneid

**Affiliations:** 1 Department of Cardiovascular Medicine, University of Texas Medical Branch, Galveston, TX, USA; 2 Department of Emergency Medicine, University of Texas Medical Branch, Galveston, TX, USA; 3 Department of Internal Medicine, University of Texas Medical Branch, Galveston, TX, USA; 4 John Sealy School of Medicine, University of Texas Medical Branch, Galveston, TX, USA

**Keywords:** diabetes mellitus, heart failure, tirzepatide, dipeptidyl peptidase-4 inhibitors, cardiology

## Abstract

While patients with type 2 diabetes mellitus (T2DM) and heart failure (HF) are frequently prescribed dipeptidyl peptidase-4 inhibitors (DPP-4is), emerging evidence suggests glucagon-like peptide-1 receptor agonists may offer improved outcomes. This study compared the effectiveness of tirzepatide versus DPP-4i in patients with T2DM and HF. Adults with T2DM and HF treated between 2022 and 2025 were included. Cohort A comprised patients receiving tirzepatide, and Cohort B comprised patients receiving DPP-4is. Prespecified subgroup analyses were conducted for HF with reduced ejection fraction (HFrEF) and HF with non-reduced ejection fraction (HFnonrEF). Propensity score matching was performed across demographic, clinical, medication, and laboratory covariates. Outcomes included all-cause mortality, hospitalization, HF exacerbation, and major adverse cardiovascular events (MACE). After matching, 8,956 patients were included in each group. Tirzepatide therapy was associated with a lower hazard of all-cause mortality (HR 0.32, 95% CI 0.25–0.42), hospitalizations (HR 0.53, 95% CI 0.48–0.57), HF exacerbation (HR 0.37, 95% CI 0.33–0.42) and MACE (HR 0.79, 95% CI 0.73–0.84). Sub-group analyses for HFrEF and HFnonrEF demonstrated similar trends. In conclusion, among patients with T2DM and HF, treatment with tirzepatide was associated with markedly lower hazards of mortality, hospitalization, HF exacerbation, and MACE compared to DPP-4 inhibitors.

## Graphical abstract

**Graphical Abstract:**
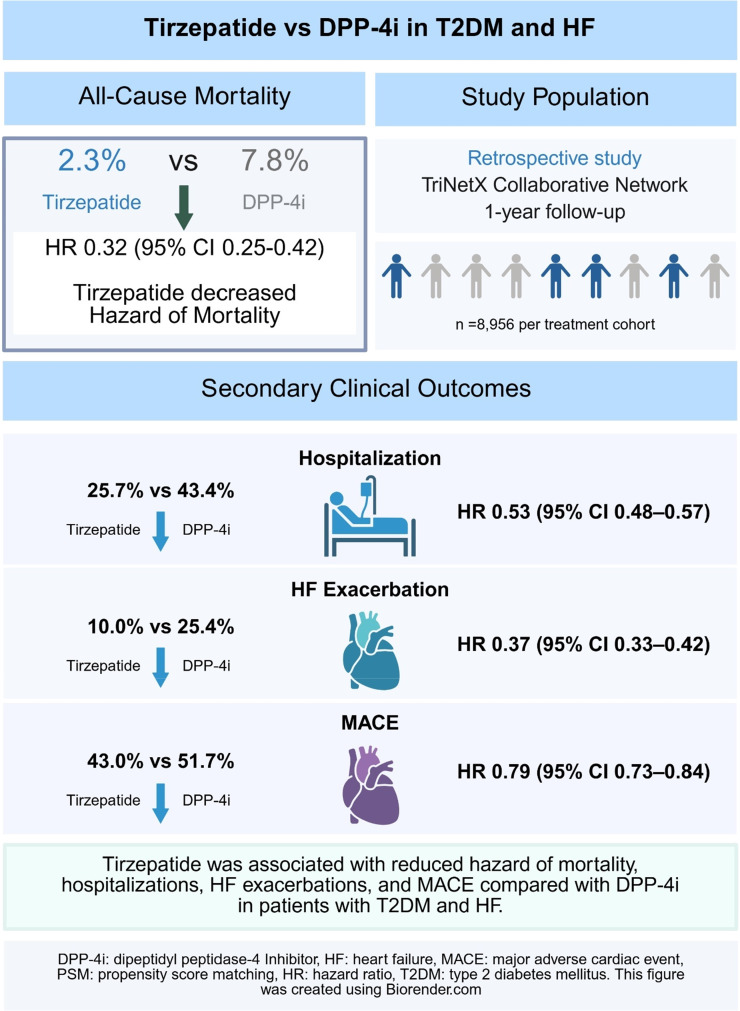

## Introduction

Heart failure (HF) affects approximately 22% of patients with type 2 diabetes mellitus (T2DM), translating to an estimated eight million affected individuals in the United States.^1^ This population disproportionately experiences high rates of emergency department utilization, hospitalizations, and mortality.^2^ Dipeptidyl peptidase-4 inhibitors (DPP-4is) and dual glucose-dependent insulinotropic polypeptide (GIP)/Glucagon-like peptide-1 (GLP-1) receptor agonists (RA) have emerged as therapeutic options with potential to reduce adverse outcomes in this high-risk group. DPP-4is prevent incretin degradation to improve glycemic control in patients with T2DM.^3^ They are commonly used as second-line therapy and are prescribed to millions of patients. DPP-4is effectively lower HbA1c and have been associated with reduced risk of major adverse cardiovascular events (MACE), as well as improvements in clinical outcomes, including fewer hospitalizations and lower mortality.^4–6^ Although DPP-4is have demonstrated clinical utility, emerging evidence suggests that dual GIP/GLP-1 RAs, such as tirzepatide, enhance incretin activity through combined GIP and GLP-1 receptor activation, leading to increased insulin secretion, glucagon suppression, and earlier satiety.^7^ These therapies have rapidly gained clinical prominence and may offer advantages over DPP-4i.

A growing body of evidence links GLP-1 RAs with superior outcomes compared to DPP-4is among patients with T2DM, HF, and renal disease.^8–10^ However, data specifically examining patients with coexisting HF and T2DM remains limited, despite the high prevalence of this population in the United States. This study addresses this gap by comparing hospitalization rates and mortality outcomes between patients treated with DPP-4i and those receiving GLP-1 RAs, specifically tirzepatide.

## Methods

**Figure 1.**
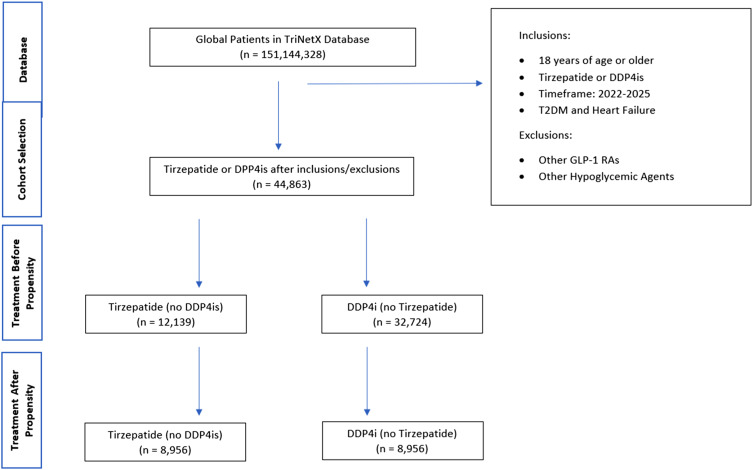
Flow chart showing the total sample size and the two cohorts after propensity score matching DPP-4is: Dipeptidyl peptidase 4 inhibitors; GLP-1 RAs: Glucagon-like peptide-1 receptor agonists; T2DM: Type 2 diabetes mellitus.

This retrospective, propensity score-matched (PSM) study utilized the TriNetX^®^ Global Collaborative Network, which aggregates de-identified patient data using electronic medical records (EMR) from 160 participating healthcare organizations (HCO), including academic medical centers, integrated delivery networks, specialty hospitals, large physician practices, and community-based healthcare providers. Adults ≥18 years of age with diagnoses of T2DM (ICD-10 E11.x) and HF (ICD-10 I50.x) who initiated tirzepatide or a DPP-4i between September 1, 2022, and November 1, 2025 were identified, and they could not have had a history of prior GLP-1 analogues nor DPP-4i use. The inclusion starting date was chosen after tirzepatide received approval for T2DM by the U.S. Food and Drug Administration (FDA). Patients with prior exposure to GLP-1 RAs or DPP4is, incomplete demographic data, or inadequate follow-up were excluded. Cohort A received tirzepatide, and Cohort B received DPP-4i (Figure 1). The index date was defined as the date of the first prescription for the respective medication. Prespecified sub-groups analyses were performed by stratifying patients by HF status into HF with reduced ejection fraction (HFrEF, EF< 40%) and HF with non-reduced ejection fraction (HFnonrEF, EF≥ 40%).

The outcomes were all-cause mortality, incident hospitalizations, HF exacerbation, and MACE assessed over 12 months from the index event. Mortality data in TriNetX are derived from EMR data and HCOs, in conjunction with the national death registries. Although deaths occurring outside the TriNetX system may be missed, this limitation is minimized by registry linkage covering approximately 94% of participating HCOs, with coverage continuing to expand.

Propensity scoring matching was performed using the TriNetX “Greedy Nearest Neighbor Matching” algorithm with a 1:1 ratio, adjusting for 41 demographic, clinical, medication, and laboratory covariates, yielding 8,956 matched patients per cohort. Covariates include age, sex, race, ethnicity, obesity, hypertension, ischemic heart disease, atrial fibrillation, hyperlipidemia, chronic kidney disease (CKD), and cerebrovascular disease. Covariates were selected a priori based on clinical relevance and prior cardiovascular outcomes literature rather than through automated or data-driven selection. Covariate balance between cohorts was assessed using standardized mean differences, with values <0.1 indicating adequate balance. A list of covariates utilized in the creation of cohorts can be found in the supplemental table.

After matching, standardized mean differences (SMD) for all covariates were <0.1, confirming adequate balance. Risk estimates with corresponding Hazard ratios (HR), 95% confidence intervals (CIs), and probability values (p-values) were generated using the TriNetX Measure of Association tool. Kaplan–Meier survival analyses were performed to compare time-to-event outcomes for mortality and hospitalizations.

This study utilized de-identified data from TriNetX® and was determined to be exempt by the Institutional Review Board (IRB) at the University of Texas Medical Branch (UTMB). The UTMB IRB determined that this project does not involve intervention or interaction with human subjects and is de-identified per the de-identification standard defined in Section §164.514(a) of the HIPAA Privacy Rule.

## Results

A total of 44,863 patients met inclusion criteria prior to propensity matching, with 12,139 patients prescribed tirzepatide and 32,724 prescribed DPP-4i. After performing 1:1 PSM, 8,956 patients remained in each cohort. Baseline demographic, clinical characteristics, medications, and laboratory characteristics were well-balanced between the two cohorts (Table 1). Patients had a mean age at index was 66.6 years with similar racial distributions and comparable prevalence of cardiovascular comorbidities, medication use, and key laboratory measures across cohorts.

**Table 1.** Baseline characteristics of patients taking tirzepatide vs DPP4i before and after PSM.

​	Before PSM				After PSM			
	Tirzepatide	DPP-4i	SMD	P-value	Tirzepatide	DPP-4i	SMD	P-value
Demographics								
Age, mean (SD)	64.0 ± 11.4	70.5 ± 11.3	0.570	<0.001	67.3 ± 10.2	67.1 ± 11.5	0.018	0.465
Female, %	46.3%	46.1%	0.003	0.858	46.5%	46.9%	0.007	0.786
White, %	74.6%	61.2%	0.288	<0.001	70.8%	70.0%	0.019	0.450
Black or African American, %	16.5%	20.0%	0.090	<0.001	17.8%	18.6%	0.021	0.388
Asian, %	1.4%	7.3%	0.291	<0.001	2.4%	2.5%	0.002	0.936
Diagnoses								
Ischemic heart disease, %	39.6%	53.2%	0.276	<0.001	42.4%	43.5%	0.022	0.370
Cardiomyopathy, %	15.2%	15.0%	0.003	0.857	12.8%	13.6%	0.023	0.344
Atrial fibrillation/flutter, %	27.4%	34.6%	0.156	<0.001	28.0%	28.1%	0.002	0.934
Old myocardial infarction, %	8.8%	13.7%	0.155	<0.001	10.6%	10.2%	0.015	0.545
Peripheral vascular disease, %	6.4%	10.0%	0.133	<0.001	7.0%	7.6%	0.021	0.394
Hypertensive heart disease, %	26.8%	32.5%	0.125	<0.001	27.9%	28.3%	0.009	0.722
Essential hypertension, %	64.8%	61.4%	0.070	<0.001	60.4%	61.4%	0.021	0.391
Hyperlipidemia, %	61.1%	67.4%	0.131	<0.001	60.7%	61.7%	0.021	0.390
Chronic kidney disease, %	29.7%	50.1%	0.426	<0.001	35.8%	36.1%	0.007	0.778
Prior stroke, %	3.7%	8.2%	0.193	<0.001	4.8%	4.9%	0.003	0.908
Laboratory Values								
BMI, mean (SD)	39.1 ± 8.2	32.2 ± 6.7	0.923	<0.001	36.4 ± 7.5	35.4 ± 7.2	0.142	<0.001
Sodium, mean (SD)	138.8 ± 3.1	137.6 ± 4.0	0.326	<0.001	138.7 ± 3.2	137.9 ± 3.8	0.227	<0.001
Potassium, mean (SD)	4.2 ± 0.5	4.2 ± 0.6	0.040	0.045	4.3 ± 0.5	4.2 ± 0.5	0.071	0.013
Creatinine, mean (SD)	1.5 ± 4.1	1.9 ± 1.8	0.142	<0.001	1.6 ± 4.2	1.6 ± 1.5	0.011	0.706
eGFR, mean (SD)	64.1 ± 27.3	52.2 ± 31.0	0.407	<0.001	59.8 ± 28.0	58.9 ± 29.6	0.032	0.258
Hemoglobin, mean (SD)	13.1 ± 2.1	11.4 ± 2.4	0.756	<0.001	12.6 ± 2.1	12.3 ± 2.3	0.151	<0.001
HbA1c, mean (SD)	7.5 ± 1.9	7.7 ± 1.8	0.114	<0.001	7.6 ± 1.9	7.8 ± 1.8	0.135	<0.001
BNP, mean (SD)	434 ± 1123	1248 ± 3576	​	​	538 ± 1298	962 ± 3911	​	​
<100 pg/mL, n(%)	434(7.4)	420(5.7)	0.067	<0.001	203(6.2)	215(6.5)	0.015	0.544
100 - 300 pg/mL, n(%)	326(5.5)	519(7.1)	0.063	<0.001	195(5.9)	211(6.4)	0.020	0.412
300 - 600 pg/mL,n(%)	176(3)	438(6)	0.144	<0.001	132(4.0)	140(4.2)	0.012	0.620
≥600 pg/mL,n(%)	191(3.2)	714(9.7)	0.265	<0.001	148(4.5)	161(4.9)	0.019	0.449
NT-proBNP, mean (SD)	1819 ± 4564	6663 ± 11579	​	​	2393 ± 5414	3586 ± 7904	​	​
<400 pg/mL,n(%)	346(5.9)	343(4.7)	0.054	0.002	163(4.9)	163(4.9)	<0.001	1
400 - 800 pg/mL,n(%)	188(3.2)	299(4.1)	0.047	0.008	110(3.3)	115(3.5)	0.008	0.734
800 - 1200 pg/mL,n(%)	106(1.8)	227(3.1)	0.084	<0.001	63(1.9)	67(2.0)	0.009	0.723
≥1200 pg/mL,n(%)	270(4.6)	1185(16.1)	0.386	<0.001	208(6.3)	214(6.5)	0.007	0.763
LVEF, mean (SD)	53.6 ± 14.1	51.7 ± 15.1	0.134	0.031	53.8 ± 13.7	51.5 ± 14.3	0.169	0.071

Abbreviations: BMI, body mass index; BNP, B-type natriuretic peptide; DPP-4i, dipeptidyl peptidase-4 inhibitor; eGFR, estimated glomerular filtration rate; HbA1c, hemoglobin A1c; LVEF, left ventricular ejection fraction; NT-proBNP, N-terminal pro–B-type natriuretic peptide; PSM, propensity score matching; SD, standard deviation; SMD, standardized mean difference.

### Main results

Over the course of 12 months of follow-up, tirzepatide use was associated with a significantly lower hazard of all-cause mortality compared with DPP-4is therapy. Mortality occurred in 2.3% of patients treated with tirzepatide and 7.8% of those taking DPP-4is (HR 0.32; 95% CI 0.25-0.42). Tirzepatide was also associated with a significantly lower hazard of hospitalization, with hospitalization occurring in 25.7% of the tirzepatide group compared to 43.4% of the DPP-4is group (HR 0.53; 95% CI 0.48-0.57). In addition, tirzepatide use was associated with a significantly lower hazard of HF exacerbation compared with DPP-4is therapy (HR 0.37; 95% CI 0.33–0.42). Similarly, tirzepatide was associated with a lower hazard of MACE relative to DPP-4is (HR 0.79; 95% CI 0.73–0.84) (Table 2).

**Table 2.** Comparative outcomes between tirzepatide and DPP-4i Therapy in patients with T2DM and HF.

​	Before PSM							After PSM						
Outcome	Tirzepatide Events (%)	DPP-4i Events (%)	ARR (%)	RR (95% CI)	OR (95% CI)	HR (95% CI)	Log-rank P-value	Tirzepatide Events (%)	DPP-4i Events (%)	ARR (%)	RR (95% CI)	OR (95% CI)	HR (95% CI)	Log-rank P-value
All-cause mortality	76 (1.3%)	258 (3.5%)	-2.2	0.190 (0.157–0.229)	0.173 (0.142–0.211)	0.20 (0.16–0.24)	<0.001	76 (2.3%)	258 (7.8%)	-5.5	0.295 (0.229–0.379)	0.278 (0.214–0.361)	0.32 (0.25–0.42)	<0.001
Hospitalization	846 (14.4%)	1,430 (19.4%)	-5.0	0.515 (0.489–0.541)	0.350 (0.325–0.377)	0.43 (0.40–0.45)	<0.001	846 (25.7%)	1,430 (43.4%)	-17.7	0.592 (0.552–0.635)	0.450 (0.406–0.500)	0.53 (0.48–0.57)	<0.001
HF exacerbation	329 (5.6%)	838 (11.4%)	-5.8	0.286 (0.262–0.313)	0.217 (0.196–0.240)	0.26 (0.23–0.28)	<0.001	329 (10.0%)	838 (25.4%)	-15.4	0.393 (0.349–0.442)	0.325 (0.283–0.373)	0.37 (0.33–0.42)	<0.001
MACE	1,416 (24.0%)	1,703 (23.2%)	0.8	0.721 (0.696–0.749)	0.529 (0.494–0.567)	0.61 (0.58–0.65)	<0.001	1,416 (43.0%)	1,703 (51.7%)	-8.7	0.831 (0.790–0.876)	0.705 (0.639–0.776)	0.79 (0.73–0.84)	<0.001

All RR, OR, and HR are expressed as tirzepatide vs DPP-4 inhibitor; values <1 indicate lower risk with tirzepatide.

Abbreviations: DPP-4i, Dipeptidyl peptidase-4 inhibitors; ARR, absolute risk reduction; RR, risk ratio; OR, odds ratio; HR, hazard ratio; CI, confidence interval; HF, heart failure; MACE, major adverse cardiac events.

### Subgroup analyses

Two subgroup analyses were conducted to evaluate the same two treatment cohorts with T2DM, stratified by HF phenotype (HFrEF and HFnonrEF). Following PSM, 204 patients were included in each HFrEF cohort, and 864 patients were retained in each HFnonrEF cohort. Baseline demographic and clinical characteristics were balanced across both sub-analyses. In the HFrEF subgroup, tirzepatide was not associated with a difference in hospitalization hazard compared with DPP-4is (HR 0.97; 95% CI 0.62-1.52). In the HFnonrEF subgroup, tirzepatide therapy was associated with a significantly lower hazard of all-cause mortality (HR 0.32, 95% CI 0.25–0.42) and hospitalization (HR 0.53; 95% CI 0.47–0.60) compared with DPP-4is therapy (Table 3).

**Table 3.** Secondary outcomes before and after PSM by HF phenotype.

Subgroup	Outcome	PSM Status	Tirzepatide Events (%)	DPP-4i Events (%)	ARR (%)	RR (95% CI)	OR (95% CI)	HR (95% CI)	Log-rank p-value
HFrEF	Hospitalization	Before	129 (37.4%)	575 (66.8%)	-29.4	0.56 (0.49–0.65)	0.35 (0.33–0.38)	0.43 (0.40–0.45)	<0.001
		After	83 (40.7%)	122 (59.8%)	-19.1	0.68 (0.56–0.83)	0.55 (0.48–0.63)	0.97 (0.62–1.52)	<0.001
HFnonrEF	All-cause mortality	Before	18 (1.2%)	247 (9.6%)	-8.4	0.13 (0.08–0.20)	0.12 (0.07–0.19)	0.20 (0.16–0.24)	<0.001
		After	12 (1.4%)	48 (5.6%)	-4.2	0.25 (0.13–0.47)	0.24 (0.12–0.46)	0.32 (0.25–0.42)	<0.001
	Hospitalization	Before	464 (31.1%)	1,566 (61.1%)	-30.0	0.51 (0.47–0.55)	0.35 (0.33–0.38)	0.43 (0.40–0.45)	<0.001
		After	282 (32.6%)	460 (53.2%)	-20.6	0.61 (0.55–0.69)	0.51 (0.46–0.57)	0.53 (0.47–0.60)	<0.001

Mortality and MACE were not evaluated in the HFrEF subgroup due to insufficient post-matching event counts.

Abbreviations: ARR, absolute risk reduction; DPP-4i, dipeptidyl peptidase-4 inhibitor; CI, confidence interval; HF, heart failure; HFrEF, heart failure with reduced ejection fraction; HFnonrEF, heart failure with non-reduced ejection fraction; HR, hazard ratio; OR, odds ratio; RR, risk ratio.

## Discussion

In this multicenter retrospective cohort study, use of tirzepatide for patients with coexisting T2DM and HF was associated with significantly lower risks of all-cause mortality, hospitalization, HF exacerbations, and MACE compared with use of DPP-4is. These findings were consistent for reduced hospitalizations across the overall cohort and EF-stratified subgroups, reinforcing a potential therapeutic advantage of dual GIP/GLP-1 RAs in this high-risk population.

Prior randomized trials have compared GLP-1 RAs such as semaglutide with DPP-4is, primarily focusing on glycemic control and weight loss in broader T2DM populations.^11,12^ In contrast, this study evaluates tirzepatide, a dual GIP/GLP-1 agonist, in a large real-world cohort of patients with established HF and emphasizes clinically meaningful outcomes, including mortality and hospitalization. Through PSM and EF-stratified analyses, our findings suggest that dual-incretin therapy may confer benefits beyond those observed with GLP-1 only agents in this high-risk population.

Recent studies, including the SUMMIT trial, have investigated the effects of dual GIP/GLP-1 therapies, particularly tirzepatide, among patients with HF, reporting a decreased risk of mortality and improvement of cardiovascular outcomes.^8,13^ Importantly, this association persisted across HF phenotypes in our study, with consistent reductions in hospitalization observed in both patients with HFrEF and those with HFnonrEF. Tirzepatide has also been associated with favorable effects on cardiometabolic risk factors, including improvements in lipid profiles, blood pressure, and inflammatory biomarkers, which are particularly relevant in HF pathophysiology.^9^ Collectively, our findings support the hypothesis that tirzepatide’s benefits extend across the HF spectrum and add to growing evidence that GLP-1–based therapies provide broader cardiometabolic advantages compared with DPP-4is.^2,4,7,10^

A plausible explanation for these findings lies in tirzepatide’s unique dual-incretin mechanism, which has been characterized as receptor-biased, with higher affinity and potency at the GIP receptor compared with the GLP-1 receptor.^14^ Prior molecular and clinical studies indicate that this dual agonism enhances insulin secretion, improves insulin sensitivity, and more effectively suppresses inappropriate glucagon secretion.^9^ Beyond its metabolic effects, the GIP RA component of tirzepatide may also exert direct effects on the vasculature that could contribute to the observed outcomes. GIP receptors are expressed on vascular endothelial cells, and GIP stimulates nitric oxide (NO) production via a calcium-mediated AMPK signaling pathway, promoting vasodilation and facilitating endothelial regeneration.^15,16^ GIP has also been shown to suppress neointimal hyperplasia in mouse models of peripheral artery disease through NO-dependent mechanisms,^15^ and to block advanced glycation end-product (AGE)-induced reactive oxygen species generation in endothelial cells.^16^ In preclinical atherosclerosis models, both GIP alone and combined GIP/GLP-1 receptor agonism attenuate atherosclerotic lesion severity, reduce macrophage-driven foam cell formation, and diminish systemic inflammation.^17–19^ Additionally, GIP and dual GIP/GLP-1 RAs lower blood pressure through mechanisms that appear to extend beyond weight loss alone, including direct vascular effects.^20^ However, the vascular effects of GIP are complex and not uniformly protective: GIP also induces endothelin-1 secretion in certain vascular beds and stimulates expression of the proatherogenic cytokine osteopontin via endothelin-1 and CREB activation, with GIP receptor expression upregulated in symptomatic atherosclerotic plaques.^21^ Furthermore, dysmetabolic conditions such as obesity and T2DM may shift GIP actions in an atherogenic direction.^22^ These tissue-specific and context-dependent vascular effects of GIP RA warrant further investigation to clarify their net contribution to cardiovascular outcomes in patients with HF. Additionally, combined GIP and GLP-1 receptor signaling contributes to clinically meaningful weight reduction, appetite regulation, and enhanced metabolic efficiency – effects that are not observed with DPP-4is.^23^ In contrast to the cardiovascular benefits demonstrated with GLP-1 RAs, multiple large cardiovascular outcome trials of DPP-4is as a class have collectively demonstrated cardiovascular neutrality, with no reduction in MACE, mortality, or HF hospitalization, and in one case (SAVOR-TIMI 53), a signal of increased HF hospitalization risk.^23,24^ These findings suggest that improved glycemic control alone does not translate into cardiovascular benefit and are concordant with the higher rates of adverse outcomes observed in the DPP-4i cohort in the present study.

In contrast, mechanistic and clinical data suggest that DPP-4is exert modest metabolic effects and does not consistently improve hemodynamic parameters or ventricular remodeling in patients with HF. Randomized trials of vildagliptin in patients with T2DM and established HF have shown no improvement in left ventricular function or clinical outcomes despite improved glycemic control, supporting a largely neutral cardiovascular profile for this drug class.^25^

Apparent discrepancies across studies may reflect differences in study design, inclusion criteria, and population characteristics. One prior analysis comparing DPP-4is with GLP-1 RAs reported a lower risk of HF hospitalization among DPP-4i users, but excluded patients with recent HF hospitalizations, thereby limiting comparability to our HF-focused cohort.^26^ In contrast, the SAVOR-TIMI trial enrolled a broader population of patients with T2DM and established cardiovascular disease or multiple cardiovascular risk factors without excluding those with baseline HF, resulting in a cohort more comparable to the present study and findings that align with our results.^27^ Together, these differences underscore the importance of population selection when interpreting comparative effectiveness studies in HF populations.

## Limitations

This study has several limitations inherent to its design, precluding causal inference. Although extensive PSM was performed across 41 covariates, important measures of diseases severity – including NT-proBNP levels, NYHA classification, and optimization of HF therapy – are incompletely captured in aggregated EMR datasets and may confound observed mortality differences. Residual confounding from unmeasured factors, including clinician decision-making patterns and socioeconomic influences, may also persist. EF-stratified subgroup analyses introduce additional considerations. While hospitalization outcomes were consistently reduced in both HFrEF and HFnonrEF subgroups, mortality could not be reliably assessed in the LVEF ≤40% cohort due to limited post-matching event counts and should be interpreted cautiously. Use of TriNetX data introduces further limitations related to coding variability, missing or underreported clinical information, and differences in documentation practices across participating HCOs. TriNetX privacy constraints also preclude assessment of clustering effects or site-level characteristics. Although mortality ascertainment is enhanced through linkage to national death registries, deaths occurring outside the network may be misses. Medication exposure was based on prescription records rather than confirmed adherence, and the 12-month follow-up may not capture longer-term differences in HF progression or survival.

## Conclusion

In this large multicenter retrospective cohort study, tirzepatide use among patients with coexisting T2DM and HF was associated with substantially lower hazards of all-cause mortality, hospitalization, HF exacerbation, and MACE compared with DPP-4i therapy. Reductions in hospitalization were observed across HF phenotypes, including both HFrEF and HFnonrEF, while mortality associations were most pronounced among patients with HFnonrEF. These findings add to growing evidence that dual GIP/GLP-1 receptor agonism may confer cardiometabolic and clinical advantages beyond glycemic control alone in this high-risk population. Tirzepatide may therefore represent a favorable therapeutic option relative to DPP-4is, although interpretation should also consider patient selection, HF severity, and access-related factors inherent to real-world studies. Prospective, randomized trials are warranted to confirm these associations, assess long-term cardiovascular and renal outcomes, and clarify mechanisms across diverse HF phenotypes.

## Supplemental material

Supplemental material - Comparative effectiveness of tirzepatide and DPP-4 inhibitors in type 2 diabetes with heart failure Supplemental material for Comparative effectiveness of tirzepatide and DPP-4 inhibitors in type 2 diabetes with heart failure by Ibrahim Mortada, Krishna Paul, Michael Chammany, Youstina Abraham, Karthik Devulapally, Aaron W. Lee, Shareef Mansour, Khaled Chatila, Michael C. Boyars, Dietrich Jehle, Thomas A. Blackwell, Hani Jneid in Diabetes & Vascular Disease Research
