# Supplementary material for: Comparative effectiveness of tirzepatide and DPP-4 inhibitors in type 2 diabetes with heart failure
Source: Diab Vasc Dis Res. 2026 Jul 8;23(4):14791641261467888. doi: 10.1177/14791641261467888 (PMC13346706; doi:10.1177/14791641261467888)
Supplement: Supplemental material - Comparative effectiveness of tirzepatide and DPP-4 inhibitors in type 2 diabetes with heart failure [file sj-pdf-1-dvr-10.1177_14791641261467888.pdf]

# Supplemental table

<!--Col Count:5-->Supplemental Table S1. Demographic, cClinical, mMedication, and lLaboratory cCovariates uUsed for pPropensity sScore mMatching

| Category | Covariate | Code System | Code(s) | Description |
|---|---|---|---|---|
| Demographics | Age at index | Derived | N/A | Age at index event |
| | Sex | HL7 | Gender:M, Gender:F | Male or Female |
| | Race - White | LOINC | 2106-3 | White |
| | Race - Black | LOINC | 2054-5 | Black or African American |
| | Race - Asian | LOINC | 2028-9 | Asian |
| Diagnosis | Type 2 diabetes mellitus | ICD-10 | E11.x | Type 2 diabetes mellitus |
| | Heart failure | ICD-10 | I50.x | Heart failure |
| | Ischemic heart disease | ICD-10 | I20–I25 | Ischemic heart diseases |
| | Cardiomyopathy | ICD-10 | I42 | Cardiomyopathy |
| | Atrial fibrillation/flutter | ICD-10 | I48 | Atrial fibrillation and flutter |
| | Prior myocardial infarction | ICD-10 | I25.2 | Old myocardial infarction |
| | Peripheral vascular disease | ICD-10 | I73 | Other peripheral vascular diseases |
| | Peripheral atherosclerosis | ICD-10 | I70.2 | Atherosclerosis of native arteries |
| | Hypertensive heart disease | ICD-10 | I11 | Hypertensive heart disease |
| | Hypertension | ICD-10 | I10 | Essential hypertension |
| | Hyperlipidemia | ICD-10 | E78 | Disorders of lipoprotein metabolism |
| | Chronic kidney disease | ICD-10 | N18 | Chronic kidney disease |
| | Prior stroke | ICD-10 | I63 | Cerebral infarction |
| Medication | Beta blockers | VA/NLM | CV100 | Beta blockers/related |
| | Angiotensin II inhibitors | VA/NLM | CV805 | Angiotensin II inhibitors |
| | ACE inhibitors | VA/NLM | CV800 | ACE inhibitors |
| | Sacubitril | RxNorm | 1656328 | Sacubitril |
| | Diuretics | VA/NLM | CV700 | Diuretics |
| | Calcium channel blockers | VA/NLM | CV200 | Calcium channel blockers |
| | Antiplatelet agents | VA/NLM | BL117 | Platelet aggregation inhibitors |
| | Biguanides | ATC | A10BA | Biguanides |
| | Sulfonylureas | ATC | A10BB | Sulfonylureas |
| | Thiazolidinediones | ATC | A10BG | Thiazolidinediones |
| | Repaglinide | RxNorm | 73044 | Repaglinide |
| | Insulin | VA/NLM | HS501 | Insulin |
| | SGLT2 inhibitors | ATC | A10BK | SGLT2 inhibitors |
| Laboratory | BMI | TNX | 9083 | Body mass index |
| | Sodium | LOINC | 9029 | Serum sodium |
| | Potassium | LOINC | 9028 | Serum potassium |

<!--Col Count:5-->Supplemental Table S1. Demographic, c~~C~~linical, m~~M~~edication, and l~~L~~aboratory c~~C~~ovariates u~~U~~sed for p~~P~~ropensity s~~S~~core m~~M~~atching

| | | | | |
|---|---|---|---|---|
| | Creatinine | LOINC | 9024 | Serum creatinine |
| | eGFR (MDRD) | LOINC | 8001 | Estimated GFR |
| | Hemoglobin | LOINC | 9014 | Hemoglobin |
| | Hemoglobin A1c | LOINC | 9037 | Hemoglobin A1c |
| | BNP | LOINC | 9003 | B-type natriuretic peptide |
| | NT-proBNP | LOINC | 9072 | N-terminal proBNP |
| | LVEF | LOINC | 2003 | Left ventricular ejection fraction |

Abbreviations: ACE, angiotensin-converting enzyme; ARB, angiotensin II receptor blocker; ATC, Anatomical Therapeutic Chemical classification system; BMI, body mass index; BNP, B-type natriuretic peptide; CKD, chronic kidney disease; eGFR, estimated glomerular filtration rate; HbA1c, hemoglobin A1c; HF, heart failure; ICD-10, International Classification of Diseases, Tenth Revision; LOINC, Logical Observation Identifiers Names and Codes; LVEF, left ventricular ejection fraction; MDRD, Modification of Diet in Renal Disease; NT-proBNP, N-terminal pro–B-type natriuretic peptide; RxNorm, normalized names for clinical drugs; SGLT2, sodium-glucose cotransporter-2; TNX, TriNetX laboratory identifier; VA, Veterans Affairs.
